# Mean flow and turbulence structure over exposed roots on a forested floodplain: Insights from a controlled laboratory experiment

**DOI:** 10.1371/journal.pone.0229306

**Published:** 2020-02-26

**Authors:** Arnold Jan H. Reesink, Stephen E. Darby, David A. Sear, Julian Leyland, Peter R. Morgan, Keith Richardson, James Brasington

**Affiliations:** 1 Lancing College, Lancing, United Kingdom; 2 School of Geography and Environmental Sciences, University of Southampton, Highfield, Southampton, United Kingdom; 3 Joint Institute for Freshwater Management, University of Waikato, NIWA, Hamilton, New Zealand; CNRS, FRANCE

## Abstract

The time-averaged and instantaneous flow velocity structures of flood waters are not well understood for irregular surfaces such as are created by the presence of roots and fallen branches on forested floodplains. Natural flow structures commonly depart systematically from those described for idealised roughness elements, and an important knowledge gap exists surrounding the effects of natural flow structures on vertical exchanges of fluid and momentum. An improved understanding of the flow structure is required to model flows over forested floodplains more accurately, and to distinguish their dynamics from non-vegetated floodplains or indeed floodplains with other vegetation types, such as reed or grass. Here we present a quantification of the three-dimensional structure of mean flow velocity and turbulence as measured under controlled conditions in an experimental flume using a physical reproduction of a patch of forested floodplain. The results conform in part to existing models of local flow structure over simple roughness elements in aspects such as flow separation downstream of protruding roots, flow reattachment, and the lowering of the velocity maximum further downstream. However, the irregular shape of the surface of the floodplain with exposed roots causes the three-dimensional flow structure to deviate from that anticipated based on previous studies of flows over idealised two-dimensional roughness elements. The results emphasise varied effects of inheritance of flow structures that are generated upstream—the local response of the flow to similar obstacles depends on their spatial organisation and larger-scale context. Key differences from idealised models include the absence of a fully-developed flow at any location in the test section, and various interactions of flow structures such as a reduction of flow separation due to cross-stream circulation and the diversion of the flow over and around the irregular shapes of the roots.

## Introduction

Floodplain topography and the structure of the vegetation on it affect the flow of flood waters during overbank events, yet few studies present measurements of mean velocity and turbulence over realistic floodplain topographies. This is a significant omission because floodplain flow is a key control on both the timing and duration of floods, such that floodplains may be used to ‘slow the flow’ [1,2]. Furthermore, overbank flow drives the deposition of sediment on floodplains and can change the dynamics of adjacent river channels [1,3–6]. Forested floodplains are abundant in both temperate and tropical climates, have great ecological value, and include some of the world’s largest floodplains, such as those that fringe the major channels of the Amazon [7–10]. Despite their significance, relatively little process-based research has been undertaken on forested floodplains, and existing conceptual and numerical models of floodplain flow processes tend to be more representative of contemporary (i.e., agriculturally modified or urban) land cover [11,12]. An improved scientific understanding of the structures of the flow over forested floodplains therefore has relevance to ecosystem conservation and restoration, flood risk management, and the interpretation of the pre-historic and geological evolution of rivers and floodplains.

**Fig 1:**
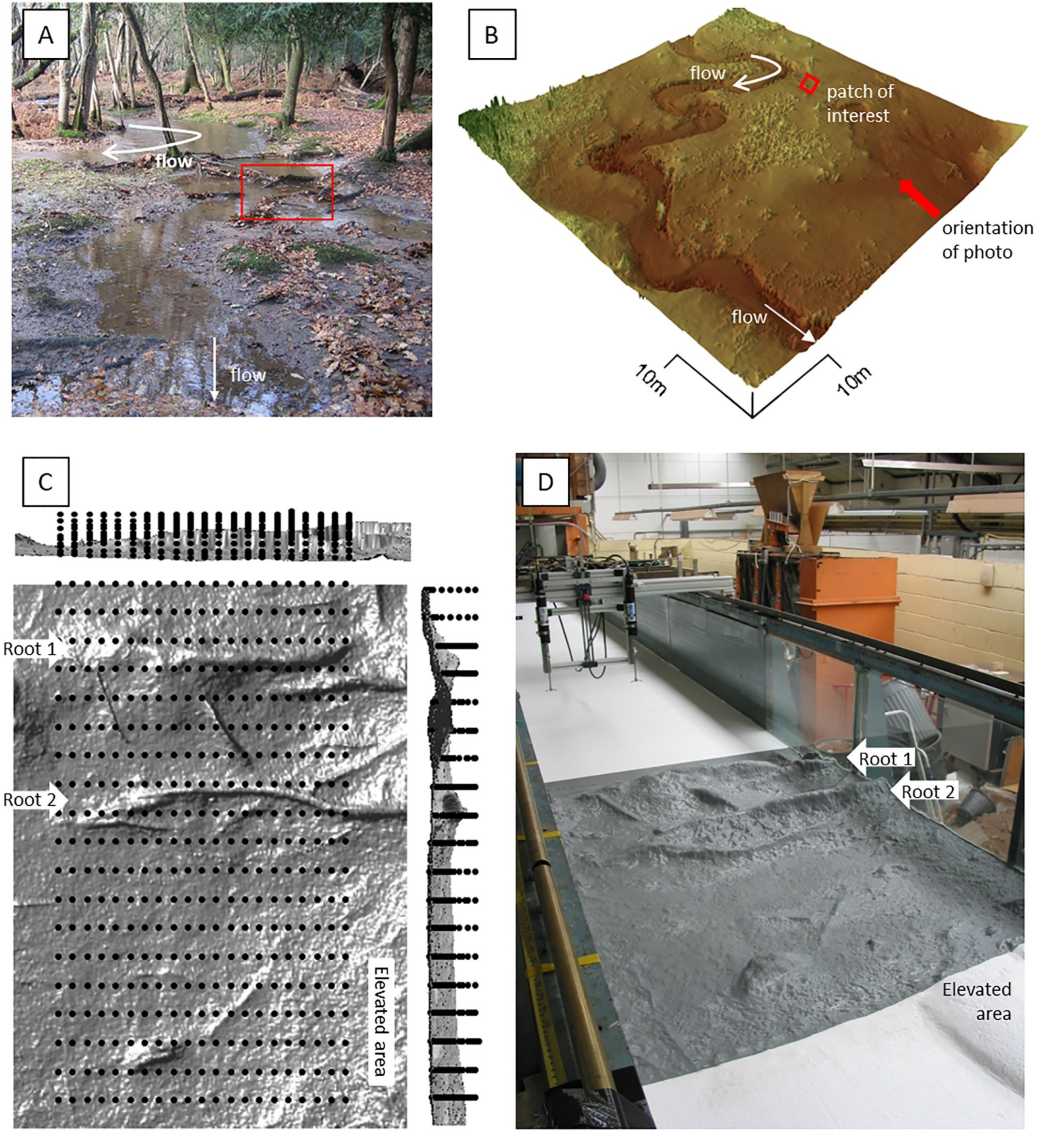
(A) Photograph of the field site showing the location of the floodplain patch scanned and replicated in this study. (B) 10-cm resolution DEM of the Highland Water study reach derived from Terrestrial Laser Scanning (TLS) data. The approximate location of the patch replicated in this study is indicated by the red box, with the orientation of the photograph indicated by the red arrow, stream flow is indicated by the white arrows and differ from the direction of floodplain flow (see also Fig 3). (C) Distribution of the ADV measurement nodes (black dots) over the 2×1.37 m area of interest. The greyscale is a hillshade such that darker areas reflect shaded (steeper) terrain. (D) Photograph of the replicated floodplain (Physical Terrain Model; PTM) and ADV instruments as deployed within the flume.

Within the specific context of forested floodplains, what little empirical research that exists highlights specific divergences from established conceptual models, in particular emphasising the complex interactions that exist between water, floodplain topography, and the assemblages of living and dead woody material that characterise their surfaces [5,13,14]. For example, previous work has identified the critical role that floodplain vegetation, topography, and organic debris play in creating spatially and temporally variable overbank fluxes of water (Fig 1A and 1B) [13,14]. Floodplain flows are relatively shallow and thus have the potential to be modified strongly not only by the floodplain topography, but also by the presence of vegetation, including exposed roots and organic debris, on the woodland floor. In such instances, flow blockage and diversion are common and conditions may also favour intense turbulence generation by both wakes and shear [15]. The magnitude of such turbulence generation may deviate from idealised models in particular because exposed roots and fallen branches are irregular in their shape, orientation, and spacing.

**Fig 2:**
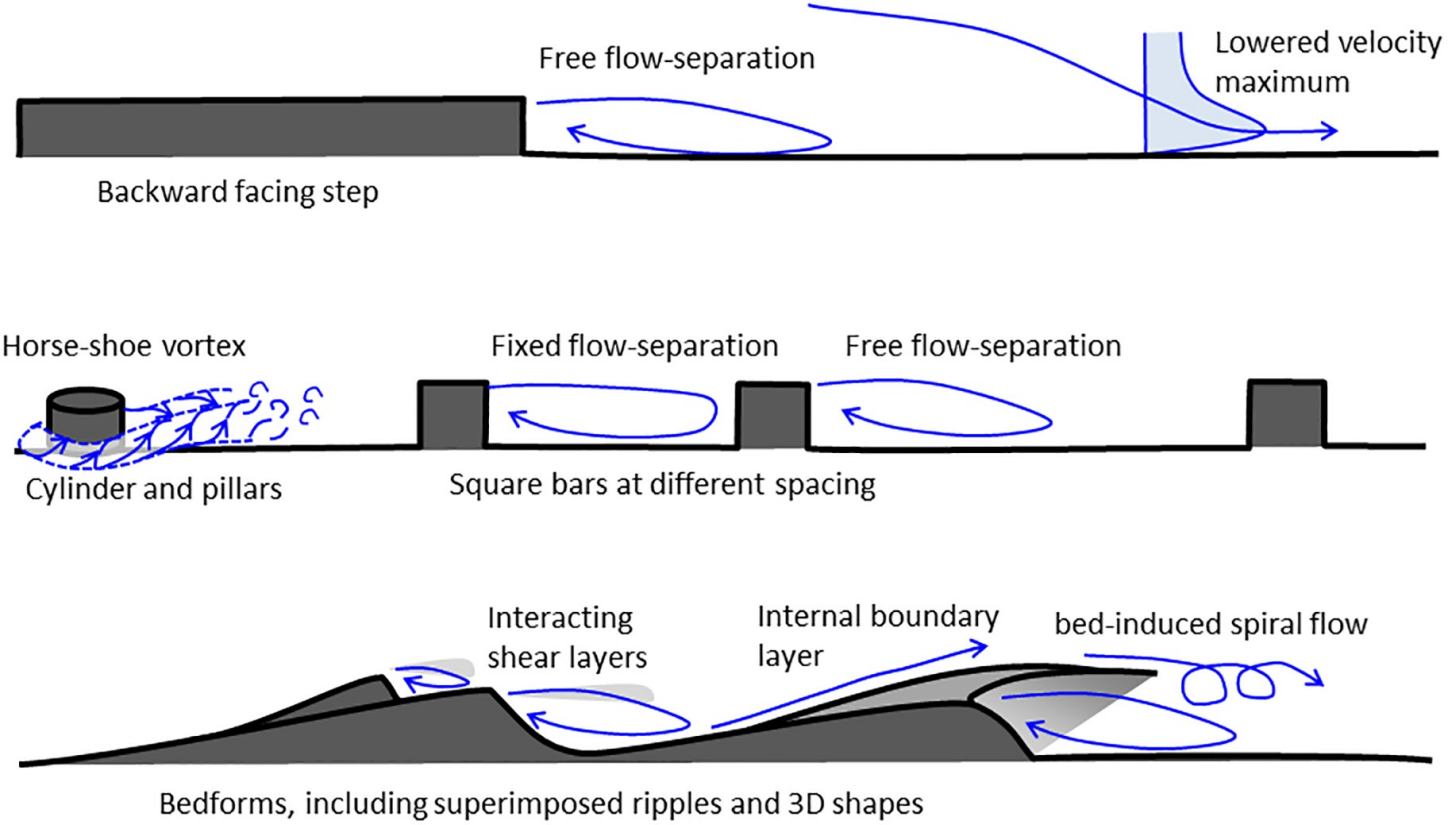
Conceptual diagram of flow structures over commonly investigated roughness elements.

The variable size, shape, and spatial organisation of roughness elements that characterise forested floodplains (Fig 1) has received little attention, even though it is known that spatial variations in roughness may change time-averaged flow velocity and turbulence [16,17]. Meanwhile, differences in the relative submergence of roughness elements are known to strongly affect hydraulic resistance [18]. Flow structures are understood relatively well for idealised bed shapes (Fig 2) such as cubes [19], square bars [20,21], cylinders [22–24], backward facing steps [25–27], and asymmetric bedforms [28–31]. These studies indicate that the presence of these different roughness elements can induce a range of secondary circulation and coherent flow structures such as separated flow cells with pronounced shear layers [32,33], lowering of the velocity maximum [25], horseshoe vortices [34,35], and secondary currents such as spiral flows [36].

Although few investigations have quantified the flow structure over distinctly irregular surfaces [37], an increasing number of investigations have tackled complex arrangements of roughness elements by changing their spacing [38–41], flexibility [42,43], porosity [44], and/or superposition [45,46]. These studies indicate that, in cases where surface roughness is distinctly heterogeneous, vertical flow velocity profiles remain underdeveloped and do not approach equilibrium. In cases where roughness elements are abundant and similar, the spacing of similar roughness elements changes overall roughness [47], with the total roughness being the sum of different sizes of common elements [45,46]. Yet, the superimposition of elements of different sizes can dramatically alter the structure of the flow [48]. The irregular, heterogeneous topography that is characteristic for forested floodplains contains elements of all these published findings, and the key question on how overbank flow responds to distinctly irregular roughness elements, such as those that characterise forested floodplains, remains unsolved.

It is apparent, therefore, that a comprehensive understanding is lacking in terms of what flow structures do occur within overbank flows on forested floodplains, and how local flow structures combine and interact within the wider flow field. Furthermore, even if process-based simulations of forested floodplain flows could be undertaken, the current lack of empirical data would prevent robust model validation. To address these gaps, in this paper we present a novel set of physical model experiments that identify the time-mean and instantaneous 3D velocity and turbulence structure of flow across an irregular experimental surface that is a direct physical copy of a natural forested floodplain. The insight on the deviations from classical models of flow structures revealed by this study is needed to guide further investigations into flow over irregular topographies, and the development of more robust models of floodplain flow in support of flood risk management.

## Aims and objectives

The central aim of this study was to acquire the flow velocity data necessary to quantify the time-mean and instantaneous 3D velocity and turbulence structures over a surface that has the topographic and vegetative complexity of a natural forested floodplain. The specific objectives were to:

1. Acquire high resolution topographic survey data from a natural forested floodplain surface;
2. Use the data from (1) to replicate a portion of the floodplain surface within a laboratory flume at 1:1 scaling;
3. Characterise the flow field over the replicated floodplain surface by acquiring 3D velocity data at high spatial resolution, and;
4. Analyse the dataset from (3) to characterise time-mean and instantaneous flow structures and develop a conceptual model of the mechanisms of turbulence production and dissipation over the experimental forested floodplain surface.

## Methods

The overall methodological strategy was to replicate over-bank flow conditions that are characteristic of field conditions, but within the controlled environment of a laboratory flume. This approach recognises that to accurately quantify the turbulent characteristics and their relationship to the underlying floodplain surface in a reproducible manner, it is necessary to acquire fully three-dimensional flow velocity data at high spatial and temporal resolution. However, such data acquisition is very challenging in the field. Therefore, we created a replica of a forested floodplain surface observed in the field within a large flume in order to achieve 1:1 hydraulic scaling that could be subject to intensive hydrodynamic measurement.

### Field study site

The study was based on a forested floodplain reach located near Millyford in the ‘*Highland Water Research Catchment”*, New Forest, UK (Fig 1A) [14,49]. Permission to undertake the fieldwork was provided by the UK Forestry Commission through a permit provided to the University of Southampton. This site was used because (i) it is a national reference site for lowland floodplain forest streams [50] and (ii) hydrological and other contextual data were readily available from related prior research on the hydrology and hydraulics of forested rivers [2,51]. We employed Terrestrial Laser Scanning (TLS) [52,53] to undertake a high resolution survey of the Millyford floodplain. Specifically, we used a Leica ScanStation time of flight system at ranges of less than 50 m, providing individual point accuracy of <2 mm, using multiple overlapping scans (±2 mm registration error) to ensure that the floodplain surface was not occluded by irregular topography and/or vegetation. The TLS data were post-processed to remove extraneous data and obvious mixed pixels (using a minimum elevation filter) using the Topographic Point Cloud Toolkit [54], to create a detailed DEM of the study reach (Fig 1B). Similar to other floodplain forests [55], the surface roughness relates principally to surficial debris, protruding roots, branches, and stems. All these features are directly incorporated as topographic features in the terrain model due to the high point density (> 10^3^/m^2^) used.

### Field to flume: Selection and reproduction of the floodplain patch

**Fig 3:**
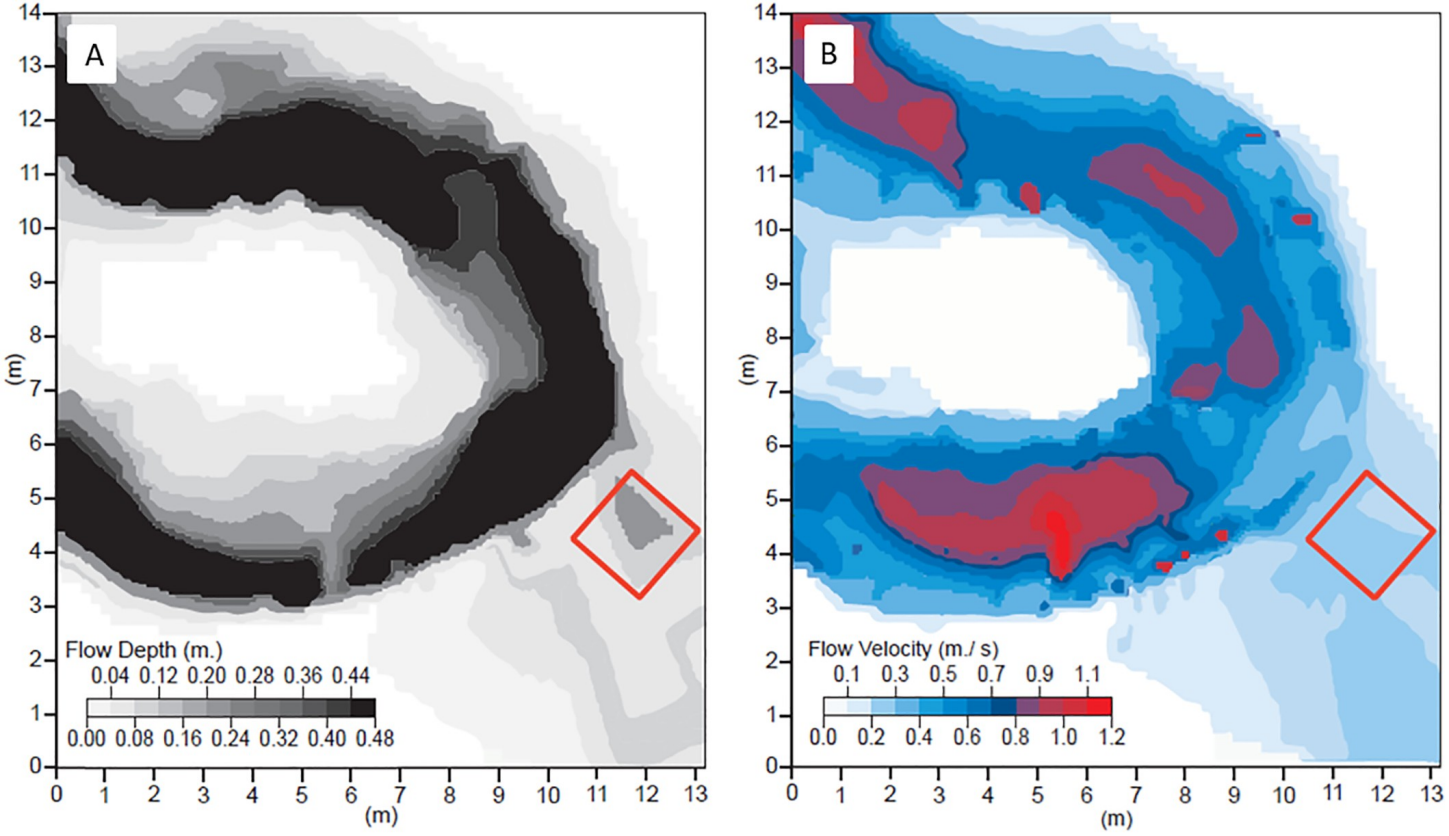
Results of Hydro2de simulations for a total flow discharge Q = 1.12 m^3^/s, showing the distribution of (left) simulated flow depth and (right) simulated flow velocity in the Highland Water study reach around the investigated floodplain patch. The flow discharge of 1.12 m^3^/s corresponds to an estimated Recurrence Interval of 6 years, based on a 5 year flow record. Model simulations were undertaken based on a 10cm resolution DEM of the reach (see Fig 1) that was acquired via TLS survey in May 2007.

We analysed the derived DEM to extract metrics which are relevant in the parameterization of flow resistance. Specifically, following [56] we employed a 3×3 moving window analysis to create a raster of surface roughness height. Within this raster we then identified an area of 2.0x1.37 m (the latter dimension being selected due to the constraint of the flume width) that contained roots and sub-metre scale topographic variability that characterises large areas of the floodplain. It is important to emphasize that the selected ‘patch’ of floodplain contains multiple roughness elements that are characteristic of the wider floodplain, but the patch itself is singular in the precise arrangement of the sizes, shapes, orientations, and spacings of its roughness elements. We do not claim that this single realisation of an irregular topography provides a representative value for forested floodplains in general, but we do argue that it makes it possible to assess to what extent we may expect flow over a realistic irregular morphology to adhere to, or deviate from, idealised models (Fig 2). The orientation of the sample patch was chosen to match the local downstream flow direction (Fig 3, red box) in order to align the roughness elements with their formative flows, and to minimize the distortion of the flow structures by the presence of flume walls relative to the structures that exist at the field site.

The floodplain ‘patch’ was used to create a 1:1 scale Physical Terrain Model (PTM) from (waterproofed, thus impermeable) high-density polyurethane using a computer-controlled milling process [57]. It should be noted that although the location of this patch was identified based on the 10 cm resolution DEM, the minimum elevation filtered scan data were used to inform the manufacture of the PTM (total of 3087 scan points within the 2×1.37 m area of interest; Fig 1C). The PTM precision is determined by the milling bit size, but since this is <1 mm, in practice the limiting factor is the precision of the scan data. The ‘DEM to PTM’ method of replicating complex topographic surfaces offers advantages over alternative techniques such as casting [58] in that it is non-invasive and avoids the use of potentially hazardous materials. We note that the use of a raster DEM means that our technique does not reproduce overhanging slopes (>90 degrees) (that is, the replicated PTM is in effect a 2.5D surface like the DEM on which it is based), however, such features were few and limited in size.

The PTM (Fig 1C and 1D) can be subdivided into three general zones: (i) two pronounced topographic highs associated with large roots that are oriented transverse to the flow direction and that are most exposed in the upstream-left of the test section, with smaller roots surrounding them; (ii) an elevated area in the downstream-left of the test section, and; (iii) a flatter area on the right-hand side of the test section. The roots are the dominant form roughness elements. The vertical distance between the top of the roots to the base of the scour in their lee varied between 0.03 and 0.1 m for the upstream transverse root (Fig 3C and 3D, Root 1), and between 0.01 and 0.1 m for the downstream root (Fig 3C and 3D, Root 2). Since the roots are located on top of an elevated section of the experimental bed, the maximum elevations of these roots protrude into the flow by as much as 61 and 75% of the mean flow depth (Fig 3C, profiles). The topography of the roots included steep slopes at the edges of the roots that exceeded 30° at 0.01 m resolution. Root 1 is composed of two off-set sections with an oblique depression between them that is 0.03–0.06 m lower than the elevated roots around it. A 0.01 to 0.07 m high, downstream-oriented, root is located between Root 1 and Root 2. The isolated obstacle in the middle of the downstream area varies between 0.01 and 0.05 m in height. The elevated area in the downstream left is 57% of the mean flow depth, and the lowest part of the downstream section is 16% of the mean flow depth: there is an overall narrowing of the cross-sectional area of the flow.

Both the floodplain surface and the protruding roots are irregular in shape, and this contrasts with analyses of flow over simplified roughness elements such as square bars, negative steps, or cylinders [25,34–35,39]. As such, the floodplain patch employed here makes it possible to compare the flow structure over the irregular floodplain surface against that expected for idealised roughness elements. However, as discussed further below, the irregular nature of the PTM prevents flow from developing an equilibrium profile. Flow separation and deceleration in the lee of obstacles is known to depend on the relative heights of the obstacles, as well as their local slopes [33,59,60]. However, it is not known whether the three-dimensionality of the roots affects the flow separation and associated presence of shear zones within the flow.

### Experimental set-up

The PTM was deployed in the University of Southampton’s Chilworth flume facility, as illustrated in Fig 1D. The Chilworth flume is a 22 m long, 1.4 m wide, and 0.6 m deep recirculating flume driven by three parallel centrifugal pumps that can achieve flow up to 0.47 m^3^ s^-1^. The flume has glass walls and can be tilted between slopes of 0 and 1:200. Discharge is monitored using two annular flow meters.

The PTM was positioned immediately downstream of a 10m-long, relatively smooth, flat-bed section over which the flow is fully developed. Employing a flat-bed section in this way reduces the legacy of upstream bed roughness elements to a minimum, which ensures that the flow structures within the test section are responding to the local roughness and ensures the reproducibility of the results.

The PTM was subjected to a steady flow discharge that matches field observations of overbank flow in the area and model output specific to the field location (Fig 3), thus achieving 1:1 hydraulic scaling. The width-depth ratio of the flow was 1:5.6 on average and allowed secondary flows to develop with minimal distortion by the side-walls. The entry flow was fully turbulent and followed a logarithmic velocity profile. We configured the experiment to correspond to an overbank flow event observed on 20^th^ October, 2004, in which the total (in-channel plus overbank) flow discharge (*Q* = 1.12 m^3^/s; as measured at a nearby gauging station) had an estimated recurrence interval of 6 years (based on a 5 year long flow record [61]) and for which flow depths on the floodplain were observed to vary in the range 0.16 < H_ob_ < 0.28 m. However, because overbank flow discharge and velocities were not measured during the event of interest, we used a depth averaged hydrodynamic model (Hydro2de [62,63]) simulation on a 0.1 m resolution grid based on the TLS data (Fig 3) to estimate the floodplain flow depth (0.25 m) and depth-averaged flow velocity (0.215 m/s) for the 20^th^ October 2004 event, and thereby guide the design of the physical model experiments. With the flume width of 1.37 m, the imposed flow depth and velocity equates to an estimated influx (i.e., overbank) discharge of 0.086 m^3^/s. This imposed overbank discharge was maintained using one of the flume’s three 150 l/s capacity pumps, with the flume tailgate being used to maintain the 0.25 m flow depth in the run-in section above the floodplain patch. The slope of the flume was set in accordance with the local floodplain gradient.

### Flow data acquisition, quality control and analysis

Flow velocity time-series were acquired over the PTM floodplain patch at each node of a dense sampling grid (Fig 1C). This sampling grid provides a sufficiently dense network of nodes to resolve strong velocity gradients. We acquired estimates of the three (u, v, w) components of flow at each of the measurement nodes using two synchronized, down-looking, Sontek 16 MHz Micro Acoustic Doppler Velocimeters (ADVs) mounted on a traversing gantry to enable precise deployment with respect to orientation, location and tilt in Cartesian space. Note that the ADVs cannot be deployed within 5 cm of the water surface, but this is not a major limitation as the un-sampled area amounts to less than 25% of the mean flow depth and the flow structures of interest are generated by terrain features at the bed. Measurements were made in the middle of the flume and away from the glass walls by at least 0.07 m.

The ADVs were controlled using Sontek’s Horizon ADV proprietary software to collect, at each grid point, an instantaneous flow velocity time series of 120 s duration, this being sufficiently long to reliably characterise the turbulent fluctuations [64] and sampling at a frequency of 25 Hz. The 25 Hz sampling frequency was selected based on pilot runs in which we found that higher frequency velocity fluctuations were insignificant, but higher sampling frequencies led to the introduction of instrument noise that began to degrade data quality. Each individual ADV time-series was quality assured to: (i) remove unreliable and erroneous points (due to instrument noise and aliasing); and (ii) replace data points removed during quality control [65]. Specifically, each time-series was filtered using threshold values of the correlation coefficient (70%) and signal-to-noise ratio (15 dB), with subsequent de-spiking and replacement undertaken following [66]. The vast majority of acquired series were found to be of high quality, as indicated by the mean (91.5%) and median (94.3%) number of points retained after filtering and de-spiking. Only a small number (3%) of the acquired time series were discarded from further analysis because of their poor quality (in these discarded series fewer than 75% of the originally acquired data points were retained after filtering and de-spiking).

The quality-assured flow velocity time-series were then analysed using bespoke MATLAB scripts. To characterize the turbulent fluctuations, we computed the Reynolds stress (τ_Re_):

$$\tau_{\text{Re}}=-\rho \overset{ˉ}{u\prime w\prime }$$ (1)

in which ρ is the fluid density (taken as 1000 kg/m^3^) and the momentum term is given by:

$$-\overset{ˉ}{u^{\prime }w^{\prime }}=-\frac{1}{\text{n}}\sum_{i=1}^{\text{n}}\left(u_{i}-U)(w_{i}-W\right)$$ (2)

where *U* and *W* are the time-mean downstream and vertical flow velocity components, respectively; *u*_*i*_, and *w*_*i*_ are the corresponding instantaneous flow velocity components, and *n* is the number of data points in each time series. The turbulent fluctuations in the downstream, cross-stream and vertical directions—*u*′, *v*′ and *w*′—are defined as:

$$u\prime ={\;u}_{i}-\;U$$ (3a)

$$v\prime ={\;v}_{i}-\;V$$ (3b)

$$w\prime =w_{i}-\;W$$ (3c)

To characterise the instantaneous turbulence structure, we identify coherent structures based on quadrant contributions to the Reynolds stress after removing a hole size of two times the standard deviation [28 (their Fig 2), 67–69]:

$${Q_{1}=(u}^{\prime }>0)\;\&\;(w^{\prime }>0)\;\&\;(u^{\prime }w^{\prime })>h|\sigma_{u^{\prime }}\sigma_{w^{\prime }}|$$ (4a)

$${Q_{2}=(u}^{\prime }<0)\;\&\;(w^{\prime }>0)\;\&\;(u^{\prime }w^{\prime })>h|\sigma_{u^{\prime }}\sigma_{w^{\prime }}|$$ (4b)

$${Q_{3}=(u}^{\prime }<0)\;\&\;(w^{\prime }<0)\;\&\;(u^{\prime }w^{\prime })>h|\sigma_{u^{\prime }}\sigma_{w^{\prime }}|$$ (4c)

$${Q_{4}=(u}^{\prime }>0)\;\&\;(w^{\prime }<0)\;\&\;(u^{\prime }w^{\prime })>h|\sigma_{u^{\prime }}\sigma_{w^{\prime }}|$$ (4d)

in which *h* is the multiplier of the standard deviations (*σ*) that dictate the ‘hole size’ below which turbulent fluctuations are considered negligible [67,70,71]. This quadrant analysis removes the velocity fluctuations close to the mean in order to reveal the dominant direction of the largest turbulent events that contribute most to the Reynolds Stress. As such, quadrant analysis is highly useful because it reveals zones that contribute most to the transfer of momentum within the flow, and effectively visualises the ejection of slow-moving fluid into the faster-moving flow above (burst), and the injecting fast-moving fluid towards the bed (sweep) [28,67,72].

Since the flow over realistic (irregular) floodplain topography is expected to differ from flows over simplified roughness elements, emphasis is placed on the three-dimensional structure of the flow rather than on the interpretation of two-dimensional profiles. Here we use traditional vertical slices alongside three-dimensional plots with streamlines and iso-surfaces of flow parameters (*U*, *V*, *W*, *Re*, Quadrants) that help visualise the three-dimensional structure of the mean flow velocity and turbulence. The iso-surface values are chosen to optimise the visibility of the three-dimensional flow structure, but are kept to the same value for the four quadrants to enable consistent inter-comparison. The zero downstream velocity iso-surface is used to illustrate the flow separation and re-circulation in the lee of the floodplain obstacles, with the zero Reynolds stress iso-surface being used to visualise where the direction of the momentum flux is reversed, and hence, where significant changes between different terms in the momentum balance equation take place.

## Results

### Three-dimensional structure of the time-averaged velocity

**Fig 4:**
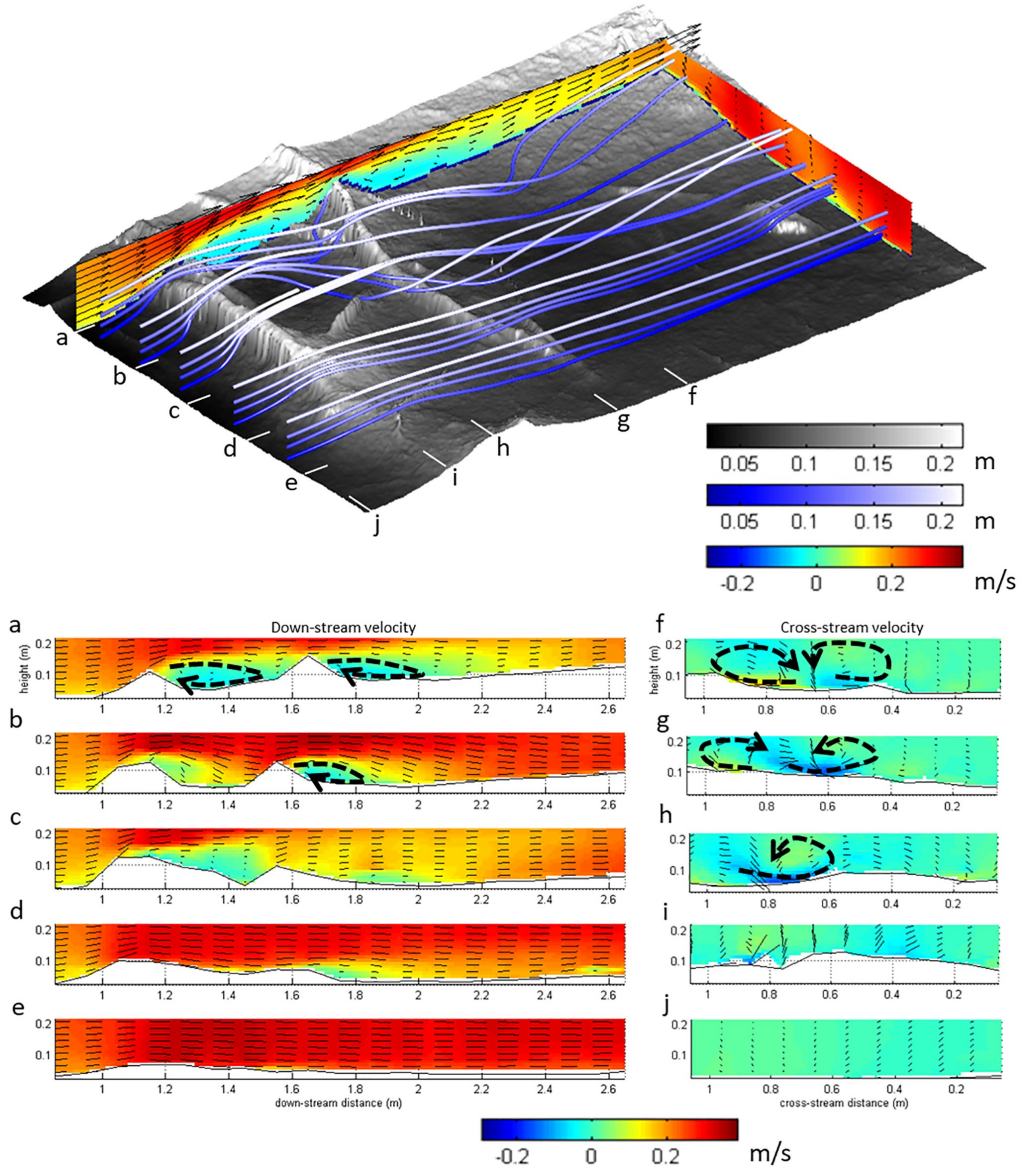
Three-dimensional structure of the flow over the floodplain patch (see Fig 1) visualised with streamlines and profiles of the downstream velocity (a-e) and cross-stream velocity (f-j). The colour-scale is the same for downstream and cross-stream velocities. Note the partial exchange of the fluid from the top and bottom of the time-averaged flow as visualised by the streamlines (coloured by elevation).

**Fig 5:**
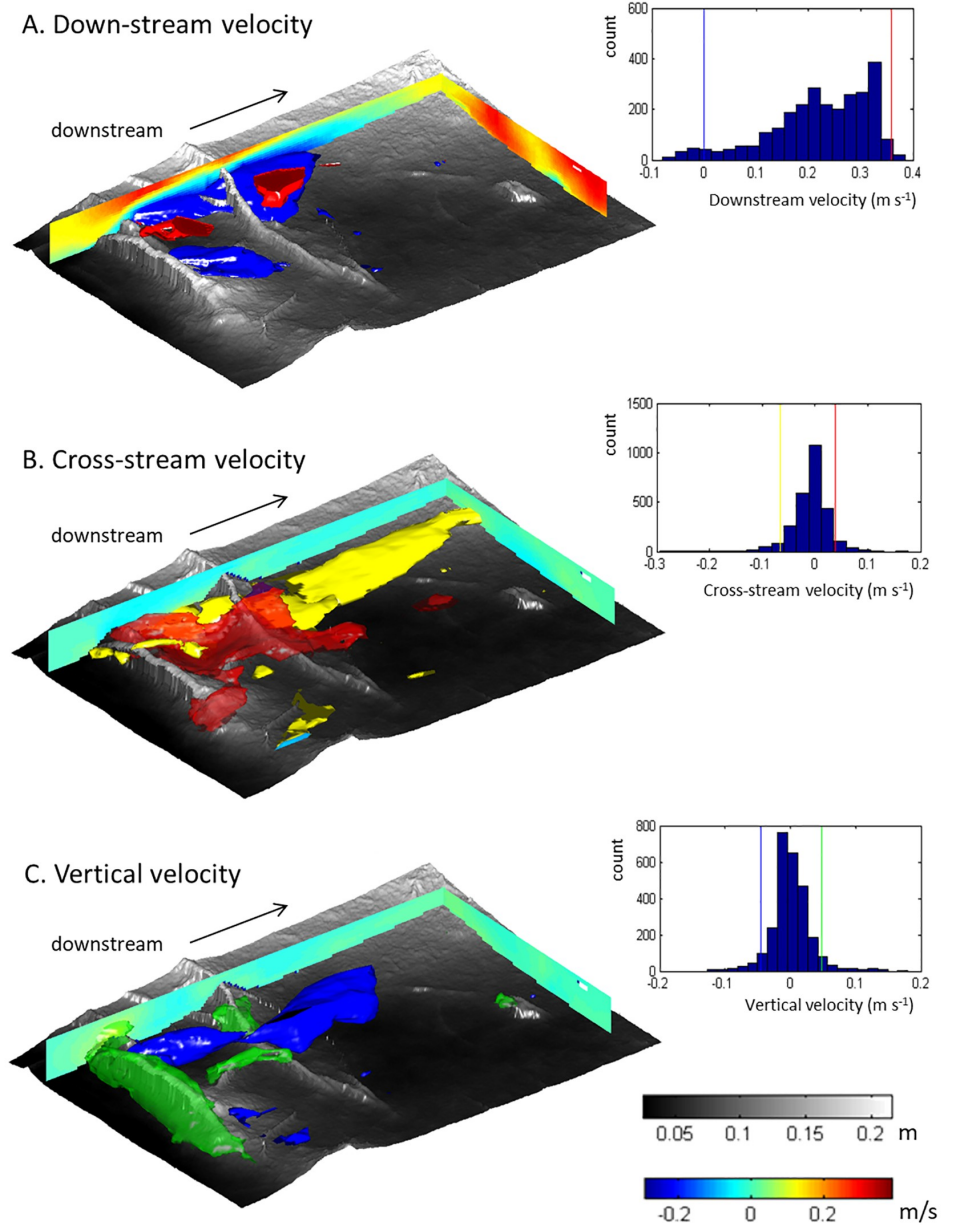
Three-dimensional structure of the time-averaged velocity field over the floodplain patch (see Fig 1) illustrated by vertical profiles and isosurfaces of selected high and low velocities. The histograms represent the velocity distributions, and the lines indicate the values of the isosurfaces. The colour-scale in the profiles is the same for downstream, cross-stream, and vertical velocities. (A) downstream velocity with isosurfaces of the recirculation zones (0 m/s, blue), and isosurfaces of the largest downstream velocities (99th percentile, transparent red); (B) cross-stream velocity, indicating flow to the left (95^th^ percentile, yellow) and flow to the right (5^th^ percentile, transparent red); (C) vertical velocity with isosurfaces that indicate the largest upward flow (95^th^ percentile, green) and downward directed flow (5^th^ percentile, blue).

The structure of the time-averaged flow field is illustrated in Figs 4 and 5. The streamlines and velocity profiles illustrate that the flow over the right side of the test section is relatively uniform (Fig 4, profile e). In contrast, the exposed roots on the left side of the test section are associated with significant three-dimensional variability in the time-averaged velocity, including flow separation and cross-stream circulations (Fig 4, profiles a-c). The scale of the flow structures over the roots is small (with typical length scales of ~0.2 m) relative to the scale of the test section. Root 1 and root 2 have distinctly different effects on the flow structure, comparable to what has been observed for square bars that are spaced at different distances (Fig 2) [20,21,39]. The arriving flow accelerates upward over the upstream root (Fig 5C, green volume), before accelerating down over and downstream of the root 2 (Fig 5C, blue volume), then splitting sideways (Fig 5B, red and yellow volumes), thus creating two prominent secondary circulation cells (Fig 4, profiles f-h), comparable to what is observed for vertical cylinders (Fig 2) [22,23,24]. Overall, the measurements highlight that the three-dimensional flow field is a combined effect of all roughness elements rather than a repetition of identical similar flow structures.

The time-averaged vertical flow velocities are highest where the flow hits root 1 (W_max_ = 0.18 m s^-1^, which is 0.8Ū, with Ū being the downstream flow velocity averaged over time and space in the test section; note that U, V and W are all plotted to the same scale). This high velocity zone matches the shape and orientation of the exposed root. In contrast, the upward velocities at root 2 (W_max_ = 0.18 m s^-1^, which is 0.8Ū) are not as pronounced as that which is caused by root 1 upstream. In terms of downwards directed flow, time-averaged vertical flow velocities are largest downstream of the root in the upper part of the flow (W_min_ = -0.13 m s^-1^, which is -0.58Ū, at z/H ≈ 0.8). In contrast to the upwards directed flow at the stoss and crests of Roots 1 and 2, this zone of downwards directed flow does not take on the shape of the root, but is instead elongated in the downstream direction and occurs above the zone where the flow over the bed splits sideways.

**Fig 6:**
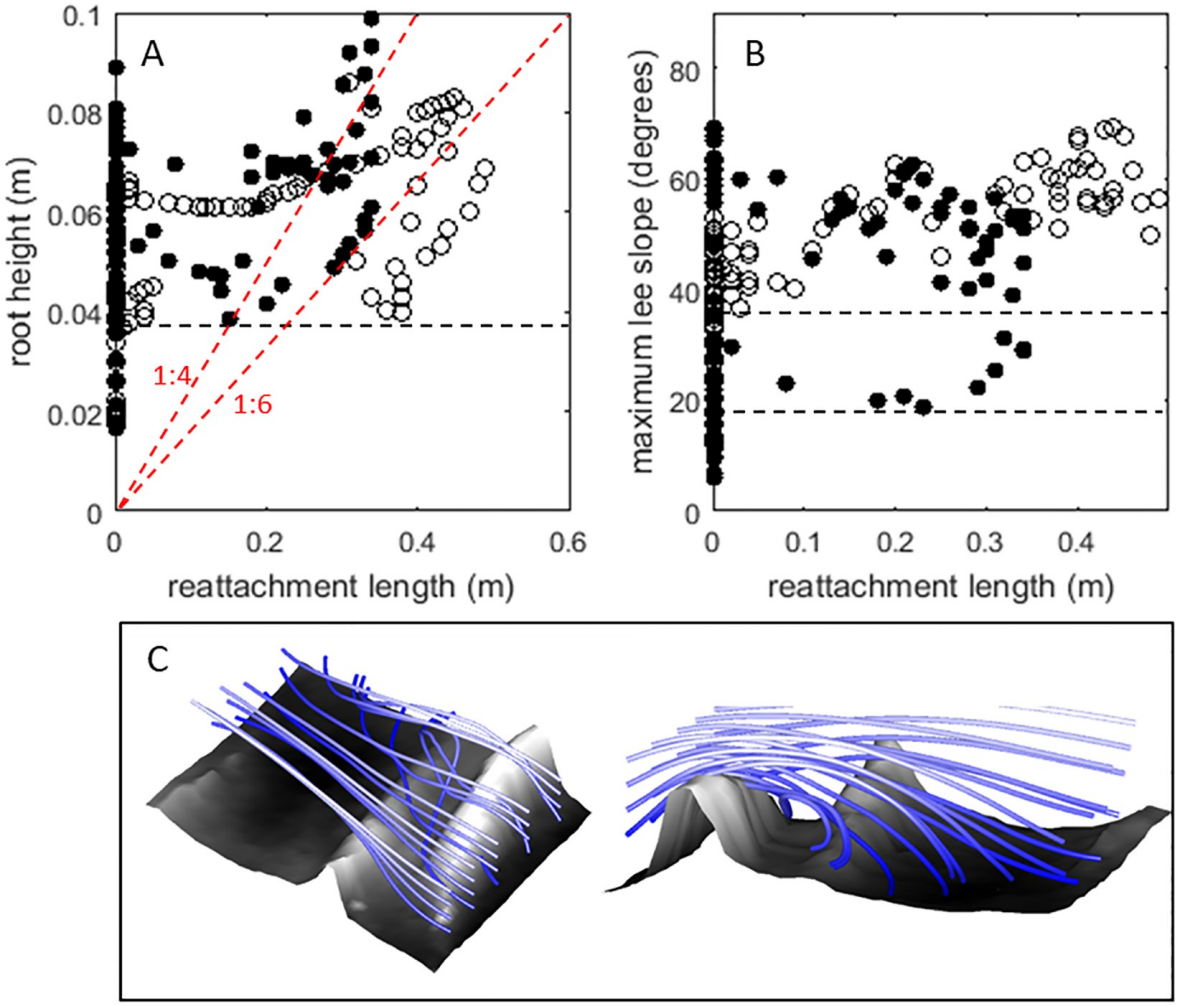
(A) Relationship between the reattachment lengths and the height of the roots. Typical relations between obstacle height and reattachment lengths (1:4 to 1:6) are indicated by the red dashed lines. (B) Relationship between the reattachment lengths and the maximum slope of negative step (left). Root 1: closed circles. Root 2: open circles. (C) Details of the flow structure over and around an oblique depression in root 1, showing strong cross-stream currents that affect the presence of flow separation (see Fig 5A, blue isosurface). Note that the black dashed lines in (A) and (B) delineate the minimum values for which flow separation was observed for the different roots.

Both the streamlines and velocity profiles highlight that the left side of the flow contains secondary circulation cells (Fig 4, profiles f-g; Fig 5B and 5C). These cells cause partial exchange of the fluid between the top and bottom of the flow, the downstream length of this secondary circulation zone being 0.5–0.8 m, which is approximately 2.2 to 3.6 times the flow depth and 5 to 8 times the maximum height of the roots. The cross-stream width of these local secondary currents is between 0.2 and 0.4 m, which is approximately 1 to 2 times the flow depth (0.2 m in this section). The maximum cross-stream velocities directed to the left generate a cross-stream current low over the bed (z/H ≈ 0.2) onto the elevated area further downstream. The maximum velocities directed to the right occur in the free stream fluid above the roots and recirculation. The largest downstream velocities (U_max_ = 0.38 m s^-1^, which is 1.7Ū; Fig 5A, red volumes) are located in the free flow downstream of the roots overlying flow separation zones (Fig 5A, blue volumes). The flow separation vortices, defined herein by their upstream flow (Fig 5A, blue volumes), occur immediately downstream from the roots in the left side of the flow, but not on the right side of the flow where the roots protrude into the flow less. Flow separation is not consistently present in the lee of all obstacles. Instead, the separated flow vortex is strongly three-dimensional in its structure and has in places significant cross-stream velocities (Fig 5A, blue volumes). Fig 6A and 6B further illustrate that flow separation is not straightforwardly dependent upon the two-dimensional geometries of the roots. Flow separation is not observed in the lee of roots less than 0.04 m high, both for the upstream and downstream root (Fig 6A), nor is flow separation observed in the lee of slopes smaller than 20° for the upstream root 1, or smaller than 40° for the downstream root 2 (Fig 6B). Thus, there is no clear trend between reattachment length and obstacle height [33] (Fig 6A, red lines) or lee slope, and flow separation is absent where it might be expected based on obstacle height and slope (Fig 6A and 6B, 0 = no flow separation). The variability in flow separation and the lack of a clear relation between obstacle height, slope, and flow separation length is attributed to the three-dimensional character of the roots and the flow, which causes the introduction of fluid into the flow separation cell from the sides (Fig 6C). Specifically, the streamlines in Fig 6C illustrate that the oblique depression in root 1 allows local cross-stream currents and spiral flows (of the kind illustrated in Fig 2) that introduce fluid into areas where flow separation would be expected based on the height and lee slope angle of the upstream root. Similar to the addition of significant hyporheic fluid in the trough of bedforms [44], the locations where flow separation is reduced or prevented are observed where lateral inflow of fluid occurs.

The flow separation in the lee of root 2 is longer than that of root 1, and the correlation between reattachment length and root height and maximum bed slope was more pronounced for the second transverse root. This variability is explained by the spacing between the roots: the flow separation in the lee of root 1 is pinned onto the second root (Fig 4A, blue volume), whereas the longer flow separation in the lee of the root 2 is free from such topographic constraint. This relation between obstacle spacing and flow separation matches observations made on square bars [39] and bedforms [40]. Thus, the results illustrate that the spacing and the geometry of the obstacles is associated with spiral flows and cross-stream currents that can change the occurrence of flow separation in the lee of obstacles.

### Vertical velocity profiles and roughness heights

The quantification of an effective roughness height for forested floodplain surfaces is complicated by the distinct irregularity of their topography. Although it is undoubtedly possible to identify effective roughness values for sufficiently large surfaces with a distribution of roughness elements, the development of a meaningful relation between hydraulic roughness and physical attributes of the floodplain, such as root height, depends on the inclusion of a sufficiently representative range of flow structures. The highly variable nature of the flow structure in this study (Figs 4 and 5) may indicate that the scale of the floodplain patch (2.5 m^2^) is too small to expect roughness values to converge.

**Fig 7:**
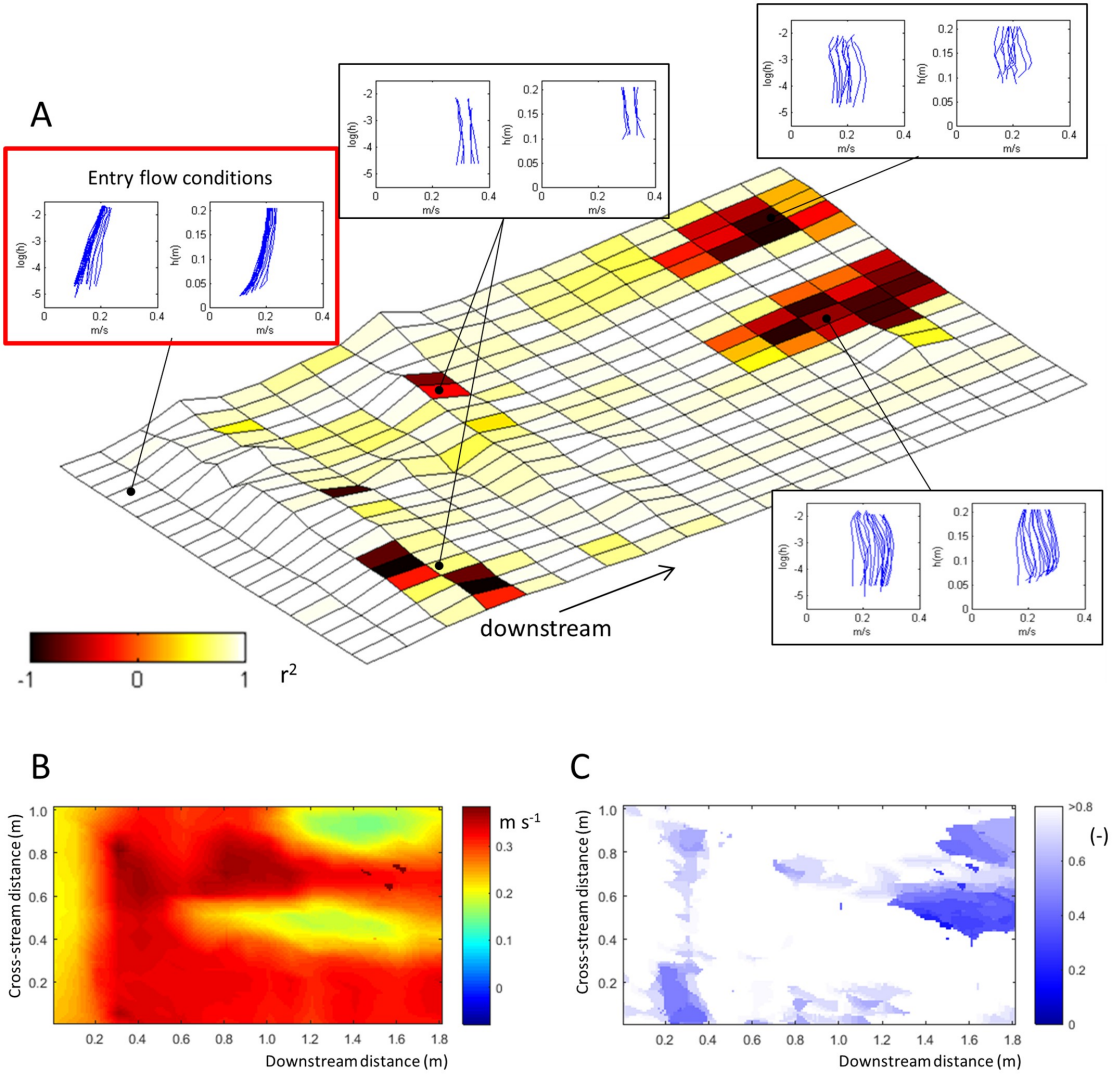
(A) Deviations from a logarithmic downstream velocity profile across the floodplain patch (see Fig 1) as indicated by the Pearson’s correlation coefficient between the downstream velocity and the log of the height above the bed: r^2^ > 0.5: good fit with logarithmic velocity profile; r^2^<0.5: significant deviation from logarithmic velocity profile. Individual log-linear and linear velocity profiles (left and right in inset plots) from key zones are plotted alongside the map. Key zones include the upstream area, which has well-established logarithmic velocity profiles (highlighted in the red box), and areas with *r*^2^ values below 0.5: over the roots; and in the zone where the velocity maximum is lowered towards the bed by secondary circulation. (B) Map of the maximum downstream velocity. (C) Elevation of the downstream velocity maximum as a fraction of the average flow depth (height/flow depth). Note that the elevation of the maximum velocity does not necessarily coincide with lower velocities: high maximum velocities may occur near the bed and low maximum velocities may occur in the free flow.

The time-averaged flow field includes a number of zones where low Pearson’s correlation coefficients indicate that the vertical profile of the downstream velocity deviates significantly from the classical logarithmic profile (Fig 7A). Whereas the inflow velocity profiles show well-developed logarithmic velocity profiles (Fig 7A, red box), as indicated by high Pearson’s correlation coefficients (r^2^; with respect to the logarithmic fit), several distinct areas further downstream in the test section have r^2^ values much lower than 0.5. The velocity profiles in these areas typically have velocity maxima that are located close (z/H ≤ 0.2) to the bed (Fig 7A and 7C). Distinct deviations from a logarithmic velocity profile near the roots are spatially restricted and associated with local flow patterns. However, two further areas with low r^2^ values exist in the downstream part of the test section. These areas coincide with injections of fast-moving fluid from the top of the flow due to the secondary currents (Figs 4 and 5), and are characterised by a low position of the downstream velocity maximum (Fig 7C) as well as lower maximum downstream velocities (Fig 7B). The vertical position of the downstream velocity maximum was also lowered locally over the upstream transverse root. Thus, exchange of the fluid from the top and bottom of the flow by secondary currents can significantly affect the shape of the velocity profile.

The calculation of a representative roughness height from time-averaged downstream velocity values based on the law-of-the-wall assumption [73,74] is affected by the spatially variable structure of the time-averaged downstream velocity. Thus, in areas where double-averaging [18,75,76] cannot be executed because of data limitations, significant uncertainty can be introduced in the analysis of the roughness height. In such cases, two strategies have in the past been applied to improve estimates of roughness heights: 1) avoidance of parts of the flow with known deviations from idealised profiles, or, 2) spatial averaging of time-averaged velocities.

**Fig 8:**
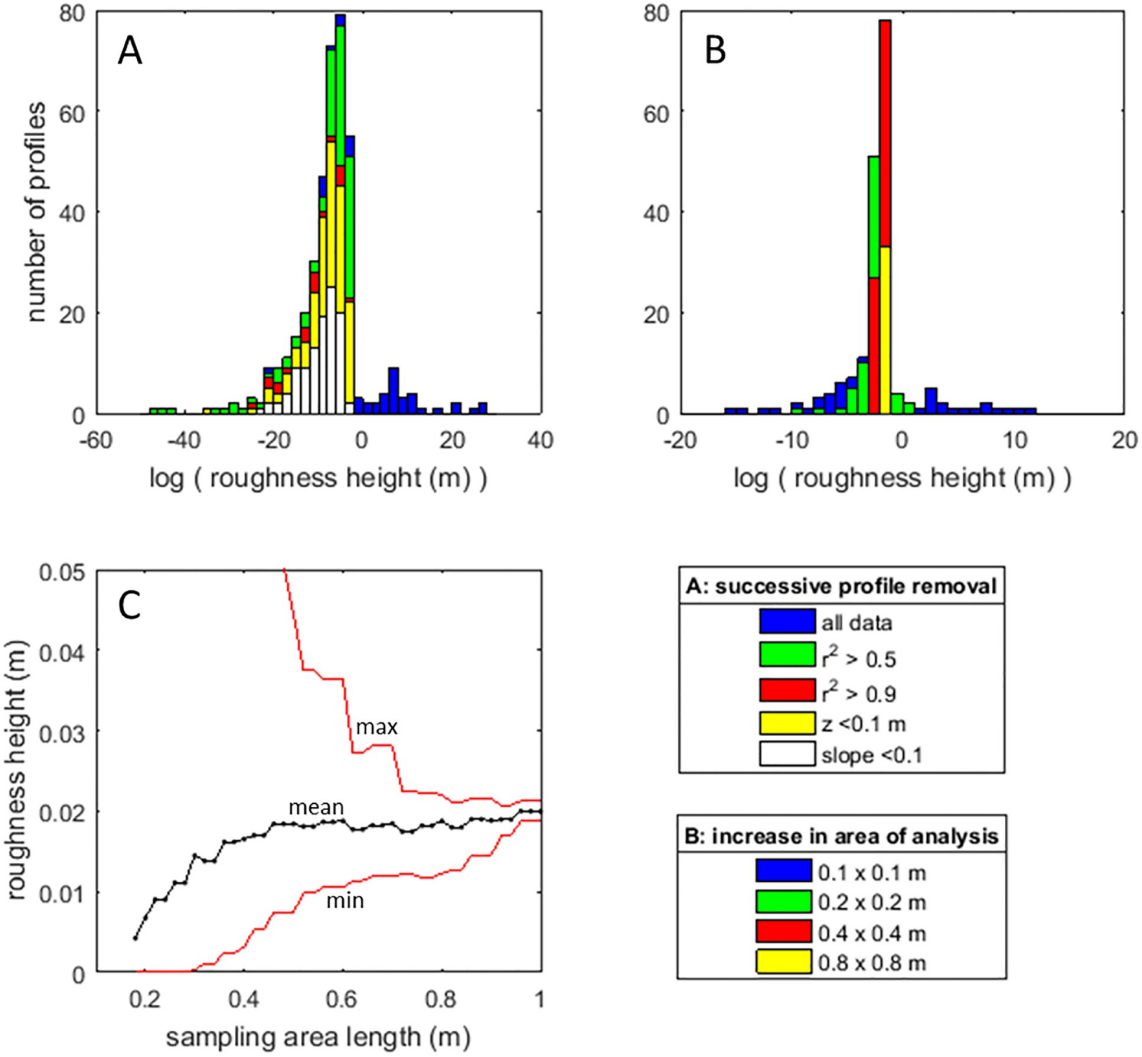
(A) Distributions of roughness height (H_0_) as calculated for individual profile. Note the strong skewness and orders of magnitude variation in the roughness height values. (B) Distributions of roughness height calculated for different sampling volumes. (C) Convergence of the roughness height values as a function of increasing the sampling volume. Note that the skewness in local roughness height values causes and initial underestimation of the average roughness height.

Selective removal of non-ideal flow may not be the best way of improving estimates of bed roughness. The data in this study show that the roughness heights calculated for individual profiles span tens of orders of magnitude, and their distribution is distinctly skewed towards very small roughness heights (Fig 8A), which appears unrealistic. To improve the calculation of roughness height, profiles can be discounted when they diverge significantly from a logarithmic profile (herein, r^2^<0.5 or r^2^<0.9 are used successively as exclusion criteria; Fig 8A), or when local bed slope and/or elevation exceed a threshold value (elevation > 0.1 m and slope > 0.1). Even then, successive removal of velocity profiles from the population based on various criteria for exclusion does not lead to convergence towards a narrow range of estimated roughness heights. Rather, the distribution maintains a negative skew and the spread in estimates of the roughness height remains large (Fig 8A). Moreover, the average roughness heights for the distributions after removal of ‘bad profiles’ range between 2×10^−8^ m to 2×10^−10^ m, which is still unrealistically small [46]. Thus, the results indicate that selective removal of velocity profiles from the dataset is not a successful strategy for the calculation of the roughness height in subcritical, shallow flow over exposed roots and branches.

Alternatively, convergence towards the average roughness height for the entire dataset (0.021 m) can be achieved by calculating roughness height based on an increasing number of velocity measurements sampled over an increasing area (Fig 8B and 8C). Prior removal of the profiles over and around the roots and the non-logarithmic velocity profiles causes a slight reduction in the roughness estimate, and can be attributed to the exclusion of the largest roughness elements as opposed to a systematic error. Thus, a roughness height estimate of 0.02 m is likely representative of the test patch as a whole, given the typical dimensions of the topographic protrusions (0.01–0.1 m) in this case.

The mean roughness height calculated for increasing surface areas increases up to sampling areas of 0.5 x 0.5 m (Fig 8C). This increase in the mean roughness value is attributed to the averaging-out of the initial skewness in the distribution. The implication of this upward trend at smaller sampling areas is that it is possible to underestimate mean roughness heights when calculating velocity profiles from high-resolution velocity datasets with similarly skewed roughness distributions. Roughness height estimates still vary within a factor of two until the area of calculation approaches the full area of interest. These findings are in line with those of studies that use the double-averaging method [18], but also emphasise that the irregular topography of forested floodplains introduces a significant error margin in estimates of the roughness height. This error margin is attributed to deviations of local velocity profiles from idealised profiles that match the local topography because of (e.g.) the inheritance of upstream flow structures. Thus, quantification of bed roughness values highlights that the roughness patch incites a unique development of the flow field rather than a repetition of comparable flow structures.

### Three-dimensional structure of Reynolds stress

**Fig 9:**
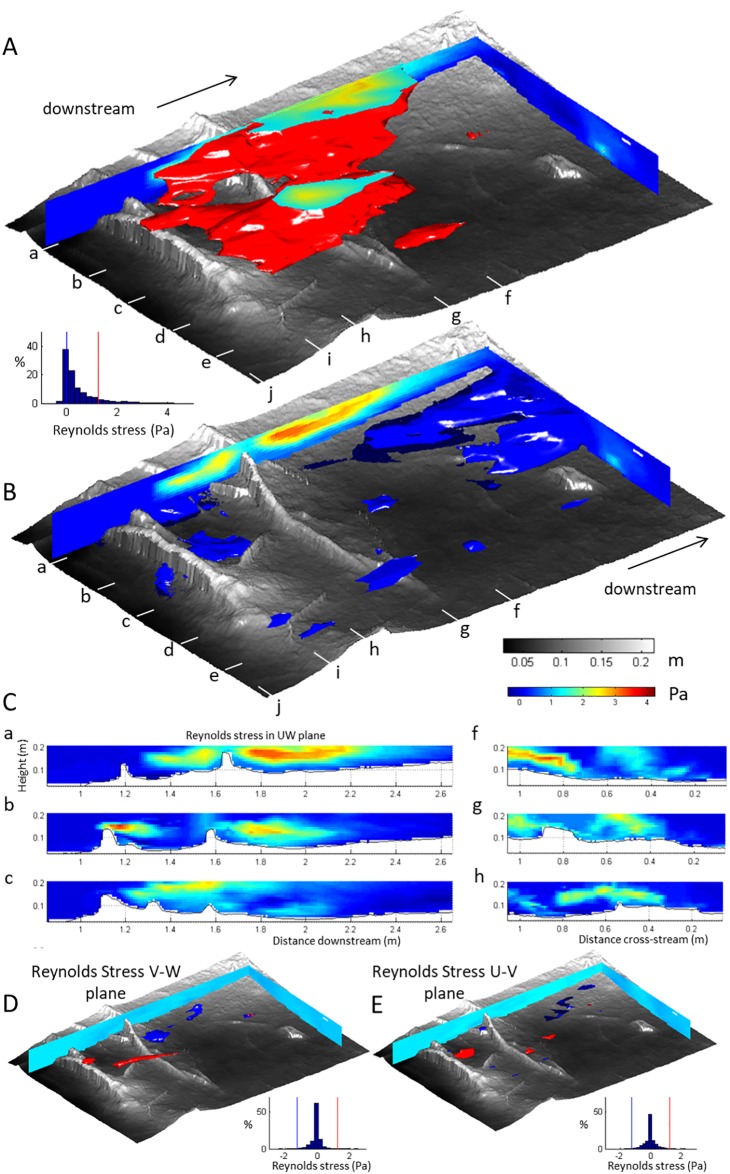
Three-dimensional structure of the Reynolds stress over the floodplain patch (see Fig 1), illustrated by vertical profiles and isosurfaces of selected values of elevated and zero Reynolds Stress. (A) Three-dimensional shape of Reynolds stress exceeding 1.25 Pa, which indicates the zones within the flow where Reynold stresses are high, is indicated by the red isosurface. At the truncation at the top of the flow, colours are interpolated following the Reynolds stress colorbar. (B) Zero Reynolds stress in the *UW* plane is indicated by the blue isosurface. The contents of the blue volume represent negative Reynolds stresses: a reversal in the direction of the momentum flux within the turbulent velocity fluctuations. (C) Downstream and cross-stream slices of Reynolds stress in the *UW* plane over the roots. (D) Three-dimensional shape of elevated and negative Reynolds stress in the *VW* and (E) *UV* planes.

The computed Reynolds stress (Eq 2) is a momentum flux that reflects the contribution of turbulent fluctuations around the time-averaged velocity to the momentum of the flow. An increase in Reynolds stress indicates a gain of momentum from turbulent fluctuations (and hence an implied loss from other terms in the momentum conservation equation). Elevated areas of Reynolds stress are located in the upper part of the flow in the central-left of the test section (Fig 9A, red volume). This zone of elevated Reynolds stress occurs downstream of the first root and is most prominent over the flow separation zones where the highest downstream velocities are also found. A gap exists in the high Reynolds stress zone just downstream from the depression in the upstream root (Fig 9A, red volume), suggesting that the local disappearance of flow separation in this area is linked to a lower production of turbulence. Nonetheless, the zones with the largest Reynolds stresses extend across both roots and indicate that the shear layers over the separated flows of the two roots are at least partially linked. Negative Reynolds stress (Fig 9B, blue volume) indicates the direction of the momentum flux is reversed in locations that are also associated with the downstream areas where flow velocity profiles have velocity maxima low above the bed. This zone occurs downstream of both roots, which indicates that this zone is influenced by the combined effect of both roots on the distribution of the Reynolds stresses. Reynolds stresses associated with turbulent fluctuations in the cross stream and horizontal planes (Fig 9C and 9D) are negligible relative to those of the *U*-*W* plane (Fig 9A and 9B).

Both the velocity profiles and the Reynolds stresses can be used to quantify bed shear stress values (e.g. Biron et al., 2004). When calculated from the velocity profiles, the distribution of bed shear stresses has a significant positive distribution with a mean value of 5.7 Pa and a median value of 1.2 Pa. However, the maximum Reynolds stress in the flow is 4.25 Pa, which indicates that the estimates from the velocity profiles are high. A second method, the extrapolation of Reynolds stress values from full vertical profiles to the bed to provide a bed shear stress value did not yield viable results for our dataset; Specifically, 47 percent of the data has a negative correlation coefficient <-0.5 as is expected for well-developed flows, 31 percent had a correlation coefficient between -0.5 and 0.5, and 22 percent exceeded a correlation coefficient of 0.5, indicating a reversal of the Reynolds stress profile caused by significant advection of turbulence into the upper parts of the flow. A third method, extrapolating only the lowest values yielded a bed shear stress of 0.26 Pa. These findings are consistent with prior work (e.g. Biron et al., 2004) in which velocity profiles are shown to produce estimates of bed shear stress around four times higher than found using Reynolds stress data. Our data add that the effect of the three-dimensional character of the flow is largest on extrapolation of the Reynolds stresses, and large on the velocity profile. The Reynolds stress nearest the bed perhaps provides the most reliable indicator of bed shear stress in comparable complex flows.

**Fig 10:**
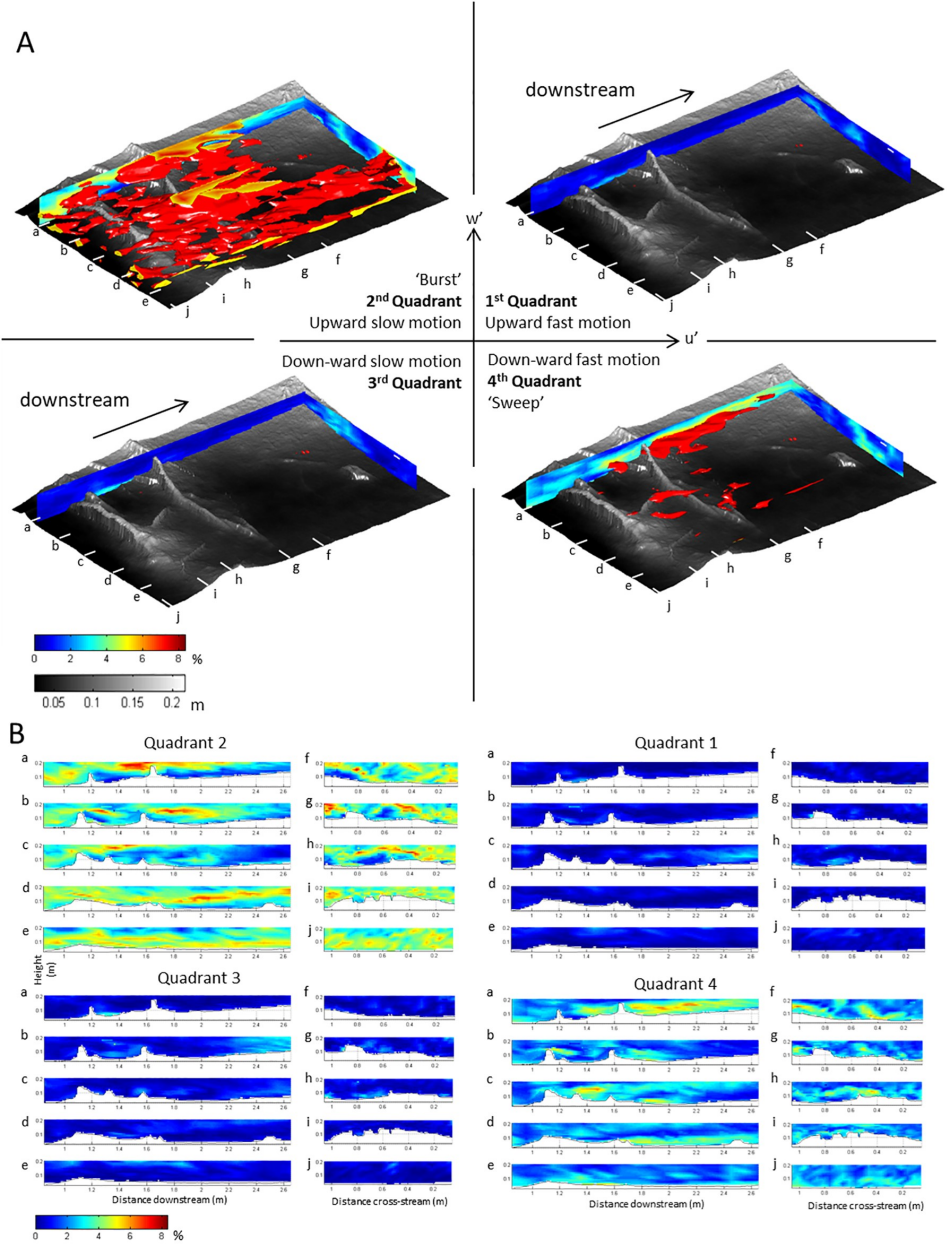
Quadrant analysis quantifies the relative contribution of large instantaneous velocities with different orientations to the overall Reynolds stress. Values are given here in percentage time that instantaneous velocities exceed of a velocity threshold value of 2σ. At the truncation at the top of the flow, colours are interpolated following the quadrant % colorbar. (A) vertical profiles and isosurfaces visualise the three-dimensional structure of the largest contributions of all four quadrants in the *UW* plane. The isosurface of the quadrant values is set to 5% (as in the colorbar) for all four quadrants; (B) Downstream and cross-stream slices of the four quadrants in the *UW* plane. Note that bursts and sweeps (quadrants 2 and 4) are dominant in their contribution to the Reynolds stress, and that the elongated shapes of the isosurfaces indicate significant advection of turbulence.

In our quadrant analysis, only velocity fluctuations in the *U*-*W* plane are analysed because the *V*-*W* and *U*-*V* planes contribute little to the overall Reynolds stress (Fig 9D and 9E). A hole size of 2σ is used to remove low-magnitude velocity events from our analysis [28,67–69,72]. Fig 10 depicts the contributions of each quadrant to the computed Reynolds stress, showing clearly that the Reynolds stress in the test section is dominated by Quadrant 2 events: bursts of slow-moving fluid that are ejected into the faster overlying flow. The bursts are particularly prominent in the upper part of the shear layer that delineates the separated flow in the lee of the roots. Quadrant 2 events are particularly prevalent in the upper part of the flow over the roots in the left side of the flow (Fig 10, profiles a-c). However, on the right-hand side of the flow, Quadrant 2 (‘Burst’) events are also dominant in the middle of the flow (Fig 10, profiles d-e). Thus, the vertical position of burst events is affected by the presence of the transverse roots and the associated flow separation cells. Quadrant 4 (‘Sweep’) events are also locally significant: sweeps being associated with fast moving fluid being injected towards the bed. Sweeps are particularly prominent in the lower part of the shear layers over the separated flow vortices. Both the zones of high Quadrant 2 and Quadrant 4 events are elongated in the downstream direction, emphasising the role of advection of the coherent flow events by the main axis of the flow.

## Discussion

### Synthesis of experimental results

**Fig 11:**
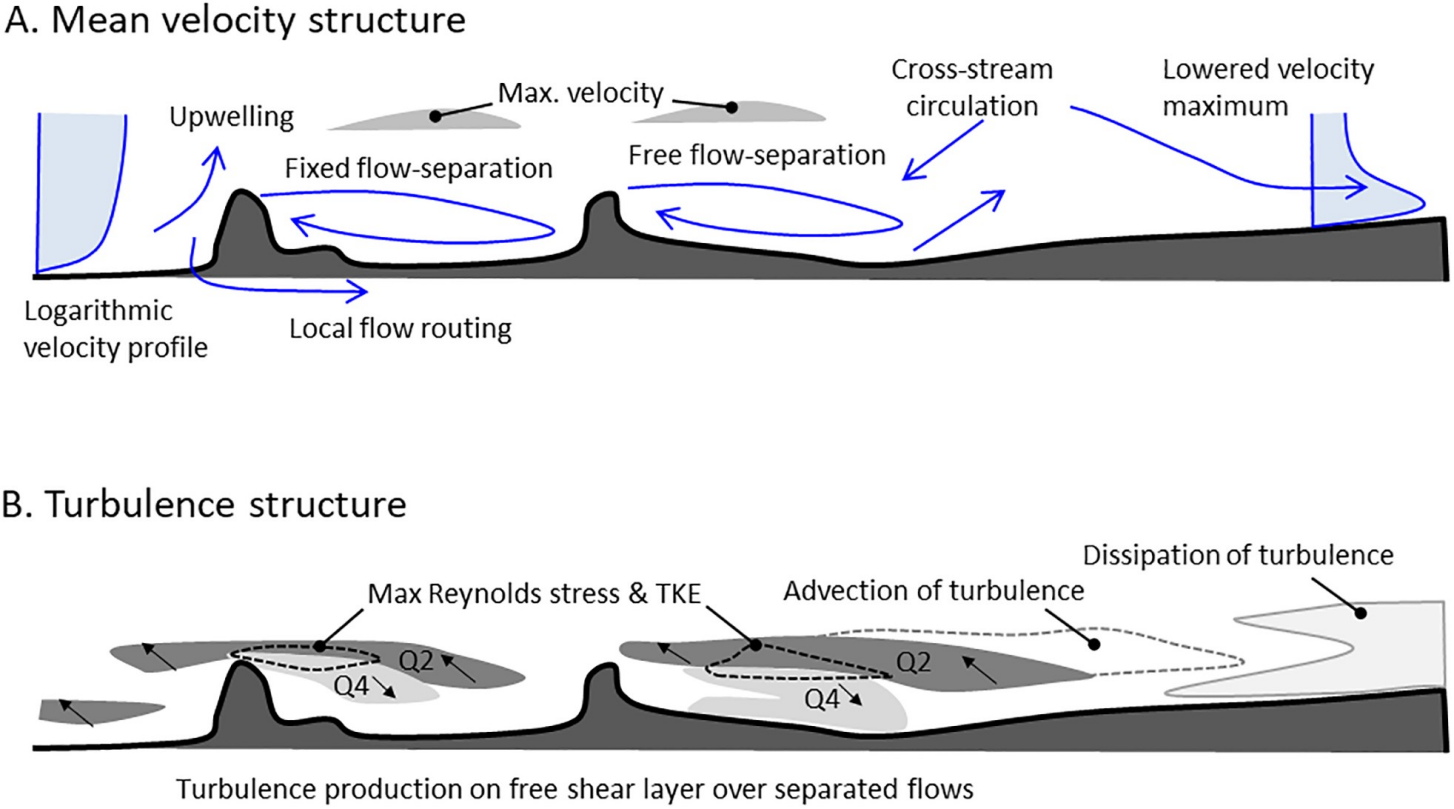
Conceptual diagram of the principal structure of A) time-averaged velocity (Figs 4 and 5) and B) turbulence (Figs 9 and 10). Q2 indicates the dominance of quadrant 2 events (sweeps) and Q4 indicates the dominance of quadrant 2 events (bursts) (see Fig 10).

The experimental results confirm the presence of flow structures that share characteristics with those observed in prior studies of idealised roughness elements such as backward facing steps [25] and square bars [39]. For example, the flow separation length in the lee of the first root is locally restricted by the presence of the second root [39,40]. These time-averaged velocity and turbulence structures are summarised in Fig 11A and 11B. The time-averaged velocity structure includes features such as: 1) local upward directed flow upstream of the roots, with significantly more upward directed flow upstream of the more exposed root 1; 2) separation of the downstream velocity at the crest of the roots, recirculation of the flow downstream of the roots, and reattachment of the flow at a distance downstream [33]; and, 3) downward displacement of the velocity maximum at a greater distance downstream that alters the velocity profile [25].

The turbulence structure includes: 1) a prominent shear layer between the recirculation zone and the overlying free flow with elevated Reynolds stress, within which Quadrant 4 events dominate the lower half and Quadrant 2 events dominate the upper half [28], and 2) a zone of negative Reynolds stresses further downstream, indicating where the direction of the momentum flux is locally reversed. The presence of flow separation is particularly important because the shear layer between the separated flow and the free flow above is associated with the greatest production of turbulence [28], because the presence of a separated flow distorts the vertical flow velocity profile and invalidates the law-of-the-wall assumption [77], and because the separated flow vortex is included in measurements of flow depth but does not contribute to downstream discharge.

### Deviations of forested floodplains from idealised flows

In addition to the flow structures described above, our data also illustrate deviations from simple flow patterns and strong spatial heterogeneity (Figs 4 and 5). The three-dimensional structure of the roots creates: 1) local flow paths (Fig 6C) that; 2) change the occurrence of flow separation in the lee of the roots; 3) initiate strong secondary circulation cells that; 4) cause slow-moving fluid to be ejected at the roots and fast-moving fluid from the free flow to be injected towards the bed downstream from the roots. Flow separation was not observed for roots smaller than 0.04 m, even though the 0.16–0.24 m long separation cells that might be expected [33] are well within the horizontal resolution of the measurements. Thus, separation of the time-averaged flow is absent in places where flow separation might be expected based on the slope and height of the roots. Where flow separation vortices were present, their lengths were mostly shorter than expected based on height-length relations typical of separated flows [33,60], with the exception of the maximum extent of the separated flow in the lee of root 2 in an area where strong secondary circulation exists. Flow separation was also not observed in the lee of slopes below 20° for root 1, and 40° for root 2. These slopes are high compared to the results of [59] and [60]. The observed deviations may be in part related to the specific spatial resolution over which the slope (0.01 m) and flow separation lengths (0.1 m) are calculated in this study, however, our measurements of reduced and shorter flow separation lengths and one locally lengthened separated flow (Fig 6), indicate significant modification and an overall reduction of the separated flow vortices. This modification and overall reduction of the separated flows is explained by the presence of strong cross-stream flows that are induced by the irregular topography of the roots. Since significant turbulence production is associated with flow separation (indicated by Q2 and Q4 events, Fig 11), the reduction in flow separation due to cross-stream circulation and local flow routing implies that the production of turbulence may also be reduced. However, the local flow paths that reduce local flow separation create lesser shear zones within the flow that, although not as pronounced as flow separation cells, also contribute to turbulence production. Furthermore, the local curvature of the flow paths is associated with centrifugal forces. The relative contributions of different terms in the momentum balance—mean velocity, turbulence, and centrifugal forces among the most prominent—is therefore expected to vary spatially across the floodplain patch in response to the topography and spacing of roughness elements.

The spatial diversity of the flow structure causes significant local deviations from an idealised logarithmic velocity profile. The areas that do not follow the ‘law of the wall’, which is a common basic assumption in hydraulic models, notably include the downstream area where the velocity maximum is located low over the bed due to the systematic downward displacement of high-velocity fluid from the free flow above (Fig 8B) [25]. The specific location where the velocity maximum lowers towards the bed is strongly influenced by the cross-stream circulation cells, and a zone of turbulence dissipation is located in the area where fluid from the upper parts of the flow is injected towards the bed by secondary circulation resulting in the lowering of the velocity maximum. Thus, the non-repetitive and irregular topography is linked to a spatially complex flow structure in which, i) turbulence production linked to local flow separation is likely reduced because of the reduction of the areas with flow separation, ii) centrifugal forces associated with tortuous flow paths are likely enhanced, and iii) turbulence may be dissipated where the velocity maximum is lowered towards the bed and the vertical velocity profiles are systematically inverted.

### Utility for future study and management of forested floodplains

The study illustrates how the irregular spacing, size, shape, and orientation of forest floor roughness elements prevent shallow overbank flows from developing a hydrodynamic equilibrium. The irregular floodplain roughness elements investigated herein create significant variability in three-dimensional organisation of both the time-averaged and instantaneous flow velocities. The irregular topography is associated with a larger-scale flow field that has a unique three-dimensional velocity structure—the total flow field is not a simple sum of its parts, but is strongly controlled by its spatial organisation. Fortunately, the structures within the investigated flow still resemble those associated with more idealised roughness elements. These findings highlight that there is an opportunity for using a Monte-Carlo or Latin Hypercube Sampling type approach [78] to the quantification of bed roughness for multiple configurations of interacting roughness elements with different sizes, shapes, orientations, and spacing.

Particular effects of the spatial organisation of the roughness elements on the flow field include the reduction of separated flows, the linking of shear layers above closely spaced obstacles, and the initiation of secondary currents that cause partial exchange of fluid between the upper and lower regions of the flow. The law-of-the-wall assumption that underpins many flood models is invalidated for underdeveloped flow conditions in which the velocity profile is not logarithmic, and this hinders the prediction of floodplain flow velocities and water depth during floods. Such a lack of uniformity within the flow may be compensated for by introducing stochastic or semi-empirical components that describe interactions of roughness elements and local steering of the flow in addition to frictional resistance.

The modification of floodplain flow by roots and fallen branches changes the spatial patterns of erosion, transport, and deposition of sediment, soil, and organic debris across the floodplain. Thus, a feedback process exists in which the floodplain structure controls its further development by sheltering its surface or exposing it to hydraulic action. Such a dynamic feedback ultimately affects the potential formation of geomorphic elements such as floodplain channels, meander chutes, floodplain debris dams, and splays. The local removal or addition of soil, seeds, and saplings has the potential to fundamentally change the structure of floodplain forest floors in ways not observed for dry woodlands. Furthermore, the effects of vegetation type and structure on floodplain development may be expected to differ between climate zones with distinct differences in vegetation type and rates of decay of fallen branches, as well as over time during the ecological maturation of floodplain forests. Thus, in addition to their ecological value, exposed roots and dead wood modify floodplain flow in a way that may impact the evolution and spatial (self-) organisation of the forest floor.

## Conclusion

This study presents a quantification of the three-dimensional structure of time-averaged and turbulent flow over roughness elements on a forested floodplain during floods. Existing models do not consider the fact that forest floodplain flows are strongly modified by the irregular topography of exposed roots on the woodland floor, such that it was heretofore unclear what flow structures exist over such forested floodplain surfaces. The key findings emerging from the results are that the irregular topography creates a flow field with a singular pattern that deviates from idealized scenarios. Notably, a significant reduction in flow separation is observed in the lee of exposed roots. The reduction of these separated flows as a consequence of local flow routing is crucial because the shear zones between the separated flow vortices and the free flow above are commonly responsible for the largest production of turbulence. The implication is that, in addition to an enhanced spatial diversity in the structure of mean velocity and turbulence, there is a potential shift in the relative contributions of different terms in the momentum balance equation, notably the relative contributions of mean flow, turbulence generated by shear in the flow and with the bed, and centrifugal forces associated with local flow routing over and around the irregular topography.

## Supporting information

S1 File Data and scripts required for reproduction of the results. (ZIP)
